# Preliminary Evaluation of Dietary Partially Hydrolyzed Guar Gum on Work Motivation and Quality of Life of Office Workers: An Open-Label, Single-Arm Study

**DOI:** 10.3390/microorganisms14102215

**Published:** 2026-10-02

**Authors:** So Morishima, Ryo Inoue, Masahiro Kawae, Ayu Suzuki, Aya Abe, Masayuki Kurokawa, Tomoko Tsuji, Tatsuo Shimosawa, Tomohisa Takagi, Yuji Naito

**Affiliations:** 1 Taiyo Kagaku Co. Ltd., 1-3 Takaramachi, Yokkaichi 510-0844, Japan; smorishima@taiyokagaku.co.jp (S.M.);; 2 Laboratory of Animal Science, Department of Applied Biological Sciences, Faculty of Agriculture, Setsunan University, Nagaotoge-cho 45-1, Hirakata 573-0101, Japan; 3 Yoshinoya Holdings Co. Ltd., 36-2 Hakozakicho, Nihonbashi, Chuo-ku 103-0015, Japan; 4 Clinical Laboratory Medicine, School of Medicine, International University of Health and Welfare, Kozunomori 4-3, Narita 286-8686, Japan; 5 Molecular Gastroenterology and Hepatology, Graduate School of Medical Science, Kyoto Prefectural University of Medicine, 465 Kajii-cho, Kamigyo-ku 602-8566, Japan; 6 Department of Human Immunology and Nutrition Science, Kyoto Prefectural University of Medicine, 465 Kajii-cho, Kamigyo-ku 602-8566, Japan

**Keywords:** partially hydrolyzed guar gum, gut microbiome, presenteeism, office worker

## Abstract

Office workers often experience gastrointestinal problems such as constipation, which can reduce labor productivity and quality of life. This study investigated the effects of continuous partially hydrolyzed guar gum (PHGG) intake on the gut microbiome, health status, and labor productivity of Japanese office workers. In this open-label, single-arm trial, 136 employees of Yoshinoya Holdings Co., Ltd. consumed 6 g of PHGG daily for two months. Outcome measures included gut microbiome composition (16S rRNA sequencing), body composition (InBody 380N), gastrointestinal symptoms (Izumo scale), sleep quality (OSA-MA), and labor productivity (WHO-HPQ). PHGG intervention was accompanied by specific genus-level alterations in the gut microbiome characterized by the enrichment of beneficial taxa including *Bifidobacterium*, *Faecalibacterium*, and *Prevotella*, despite no significant overall shift in beta-diversity. Notably, exploratory subgroup analysis indicated shifts in enterotypes from profiles previously associated with disease risks toward those more prevalent in healthy populations. Physiologically, whole-body phase angle, an indirect parameter associated with cellular status and fluid distribution, slightly increased during the study period. Furthermore, favorable changes were observed in absolute presenteeism, gastrointestinal symptoms and subjective sleep quality. Although verification with a more robust study design is necessary, these findings suggest that PHGG may be associated with positive shifts in the well-being and labor productivity of office workers.

## 1. Introduction

Office workers are exposed to specific health risks, including prolonged sedentary behavior, physical inactivity, mental stress, and irregular dietary habits, leading to increased rates of metabolic, cardiovascular, and mental health problems [1,2,3]. In particular, diminished work motivation and a decline in overall Quality of Life (QOL) are critical issues that affect not only individual well-being but also labor productivity [4,5]. Moreover, these issues are directly linked to reduced organizational productivity and consequent economic losses. Presenteeism is defined as the state of attending work while experiencing physical or psychological distress, thereby preventing full performance [6,7]. According to estimates by Nagata et al., presenteeism accounts for the largest proportion (approximately 64%) of health-related costs for companies, generating far greater economic losses than absenteeism, outpatient care, or pharmaceutical expenses [6]. Consequently, promoting the physical and mental health of workers is crucial not merely for health outcomes but also from an economic perspective.

Gastrointestinal symptoms, such as diarrhea and constipation, are frequent problems among office workers [8]. Studies have suggested that sedentary work, mental stress and poor sleep quality are associated with gastrointestinal symptoms [9,10]. Thus, the gut environment may be a contributing factor to presenteeism in this population [8,11]. Furthermore, gastrointestinal issues during working hours not only significantly impede labor productivity but may also influence their mental health via the gut–brain axis [12]. Given this context, there is a need for simple, effective interventions to improve the gut environment that can be easily incorporated into the daily routines of office workers.

To address this gap, we conducted a preliminary clinical trial to investigate the effects of partially hydrolyzed guar gum (PHGG), a widely used prebiotic dietary fiber, on the health status and labor productivity of office workers. Furthermore, we also examined how the efficacy of PHGG intervention varies depending on the subject’s enterotype [13].

## 2. Materials and Methods

### 2.1. Study Design

This study was designed as an open-label, single-arm trial and conducted at the headquarters of Yoshinoya Holdings Co., Ltd. (Tokyo, Japan) from January to March 2025. Taiyo Kagaku Co., Ltd. (TKC, Yokkaichi, Japan) prepared the study protocol and provided the test food. This study was conducted in accordance with the ethical principles based on the Helsinki Declaration and the Ethical Guidelines for Human Medical Research. A fair review was conducted from the perspective of protection of human rights and safety assurance, and the study protocol was approved by the TKC Ethics Review Committee (approval number: 2024-02A, Date of approval: 25 December 2024) and registered at the UMIN-CTR (Trial ID: UMIN000056684). The protocol was not modified from the time of the final setup or during the study. This study is reported in accordance with the TREND (Transparent Reporting of Evaluations with Nonrandomized Designs) statement.

### 2.2. Material, Supplementation, and Dosage

The test foods were PHGG (Sunfiber^®^, manufactured by TKC, Utsunomiya, Japan), and the daily dosage was set at 6 g, which contains 5 g of water-soluble dietary fiber, as measured by the Association of Official Agricultural Chemists (AOAC) method. This dosage was selected based on results from prior studies using the same daily amount [14,15,16].

### 2.3. Study Participants

Participants were recruited from the employees of Yoshinoya Holdings Co., Ltd. The inclusion criteria were as follows: (i) age between 20 and 65 years old at the time of informed consent; (ii) males and females; (iii) those who received sufficient explanation regarding the objective and summary of the trial, voluntarily decided to participate after understanding the purpose, and provided the written agreement. The exclusion criteria were as follows: (i) those with a current or past history of serious illness. In addition, severe obesity (BMI ≧ 35) was established as a pre-specified analytical exclusion criterion prior to statistical processing to prevent severe confounding effects across overall trial outcomes. The target sample size was 100, in consideration of the number of employees who could commute to the headquarters. Because this was a preliminary exploratory study, a formal statistical power calculation was not performed.

### 2.4. Intervention and Outcomes

All participants were instructed to take PHGG every morning for two months. The primary outcome was the gut microbiome composition, comprehensively evaluated through alpha-diversity (richness and evenness), beta-diversity (overall community structure), and genus-level relative abundance. The secondary outcomes included body composition assessed using InBody 380N (InBody Japan Inc., Tokyo, Japan) [17], labor productivity, quality of life (QOL), and dietary intakes (food frequency questionnaire; FFQ) [18] evaluated by questionnaires. The measurement time points for each outcome were as follows: gut microbiome: pre (baseline) and month2; body composition, labor productivity and QOL: pre, month1 and month2; dietary intake: pre. Participants were requested to attend each examination to the extent possible without interfering with their regular duties. Throughout the study period, participants were instructed to maintain their lifestyles, to avoid extreme dieting, to record lifelog data, to report any health changes promptly, and not to do excessive exercise before the measurement.

### 2.5. Labor Productivity and QOL Questionnaires

Absolute presenteeism and relative presenteeism were calculated from the WHO Health and Work Performance Questionnaire (WHO-HPQ, Japanese version) [19]. Subjective feelings related to labor productivity were rated on a visual analog scale-like scale of 0 (poor) to 100 (best) in increments of 1 for the four items: “concentration during work over the past four weeks”, “sleepiness during work over the past four weeks”, “motivation for work over the past four weeks”, and “fatigue over the past four weeks”.

QOL was evaluated using a questionnaire based on the Oguri-Shirakawa-Azumi sleep inventory MA Version (OSA-MA) and Izumo scale [20]. OSA-MA consists of 16 questions assessing 5 factors of sleep quality (“sleepiness on rising”, “initiation and maintenance of sleep”, “frequent dreaming”, “refreshing”, and “sleep length”). Izumo scale consists of indicators for abdominal symptoms such as heartburn, gastralgia, fullness, constipation and diarrhea. Participants received and answered these questionnaires via smartphones.

### 2.6. 16S rRNA Sequencing

Fecal samples were collected using a fecal collection brush filled with guanidine thiocyanate solution (Techno Suruga Lab, Shizuoka, Japan). Bacterial genomic DNA isolation, library preparation, sequencing on a MiSeq platform (Illumina, Tokyo, Japan), sequence data analysis, and a support vector machine–based classification of Japanese enterotypes were conducted as per Inoue et al. [21]. The raw 16S rRNA gene sequencing datasets generated in this study have been deposited in the NCBI Sequence Read Archive under BioProject accession number PRJNA1521603.

### 2.7. Statistical Analysis

Unless otherwise noted, all data are provided as means ± SE. Longitudinal data were analyzed using a linear mixed-effects model (LMM) with time as a fixed effect and subject as a random effect. For comparisons between groups, we employed a linear model (LM, mathematically equivalent to Analysis of Variance [ANOVA]). Model normality assumptions were verified by examining residual Q-Q plots. Data violating normality assumptions were subjected to Yeo–Johnson transformation. Post hoc comparisons for LMM and LM were adjusted using Tukey’s method. If normality assumptions remained unmet after transformation, a non-parametric Friedman test was used, followed by the Conover test with Bonferroni correction for pairwise comparisons. The *p*-value < 0.05 was considered significant. The R package MaAsLin2 was used to identify bacterial genera with significantly (q value < 0.10) changed relative abundance [22]. To explicitly account for the longitudinal, paired within-subject structure, participant ID was included as a random effect, while time point was set as a fixed effect with baseline as the reference level. The input genus-level relative abundance data were log-transformed without additional normalization. Correlations between the relative abundance of gut bacteria and the study outcomes were evaluated using Spearman’s rank correlation test. To mitigate the risk of false-positive associations arising from multiple testing, correlation analyses were strictly restricted to the 12 bacterial genera that exhibited mean relative abundances > 0.1% and significant longitudinal changes (MaAsLin2, q < 0.10). Given the preliminary and hypothesis-generating nature of this exploratory study, unadjusted *p*-values (*p* < 0.05) were utilized. All statistical analyses were performed using R statistical software (version 4.5.1).

## 3. Results

### 3.1. Subject Characteristics and Body Composition Analysis

**Figure 1:**
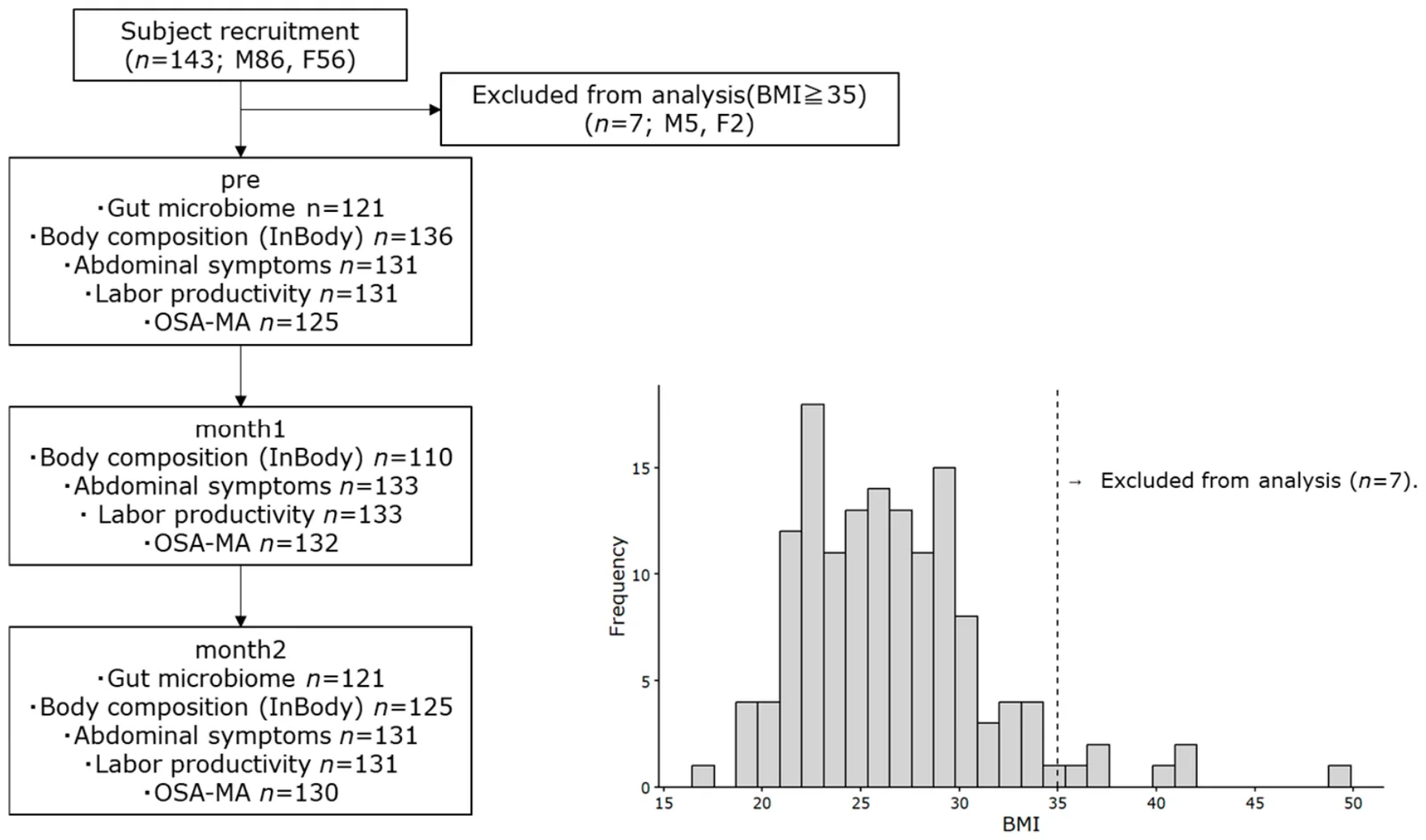
Study flow chart and BMI distribution of participants. The variation in the number of subjects across outcomes was due to occasional non-submission of fecal samples or scheduling conflicts with their regular work duties.

A total of 143 subjects participated in this study. Seven participants with severe obesity (BMI ≧ 35) at baseline were excluded prior to data analysis to eliminate potential confounding effects on data interpretation, as severe obesity markedly alters systemic metabolic baseline, bioimpedance measurements, and baseline microbial community structures (Figure 1) [23]. Although there were no dropouts, some participants missed measurements due to work-related or personal reasons; the number of subjects for each test item is shown in Figure 1. To evaluate potential attrition bias, baseline demographics, gastrointestinal symptoms, sleep quality, and labor productivity scores were compared between participants who completed the month1 measurements (*n* = 110) and those who missed them (*n* = 26). No statistically significant differences were observed across any parameter (Supplementary Table S1). Table 1 shows the characteristics of the analysis population and changes in body composition during the study period. The subjects were predominantly middle-aged, with a mean age of 48.37 years, and showed a slight tendency toward obesity, with a baseline BMI of 25.93. Most body composition parameters did not show significant changes after the two-month intervention. However, whole-body phase angle (PhA), an indirect bioimpedance parameter associated with cellular status, significantly increased both after one and two months of intervention (*p* < 0.05). Regarding safety, no severe adverse events or unintended gastrointestinal side effects related to the PHGG intervention were observed throughout the study period.

### 3.2. Microbial Composition and Digestive Status

**Figure 2:**
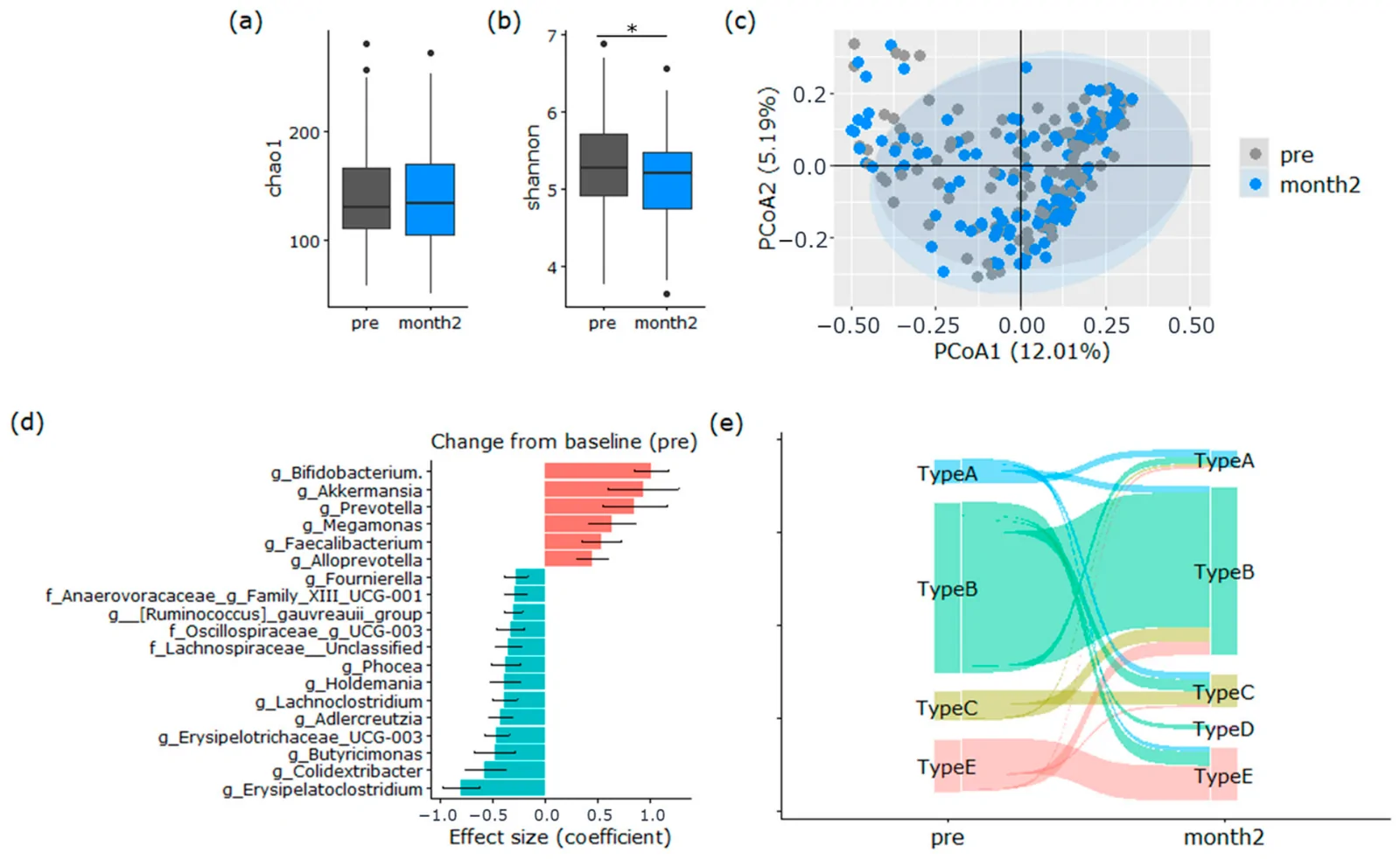
Change in gut microbial composition from baseline (pre) to month2. (**a**) chao1 and (**b**) Shannon diversity indices. * *p* < 0.05 by LMM. (**c**) Principal coordination analysis based on the Bray–Curtis dissimilarity index. The ellipse enclosing each cluster indicates a 95% confidence interval. *p* > 0.05 by permutational ANOVA test. (**d**) Bacterial genera significantly (*q* < 0.10) increased (red) and decreased (blue) in abundance after the study period. Effect size and q value were calculated by the R package Maaslin2. (**e**) Change in enterotype.

**Figure 3:**
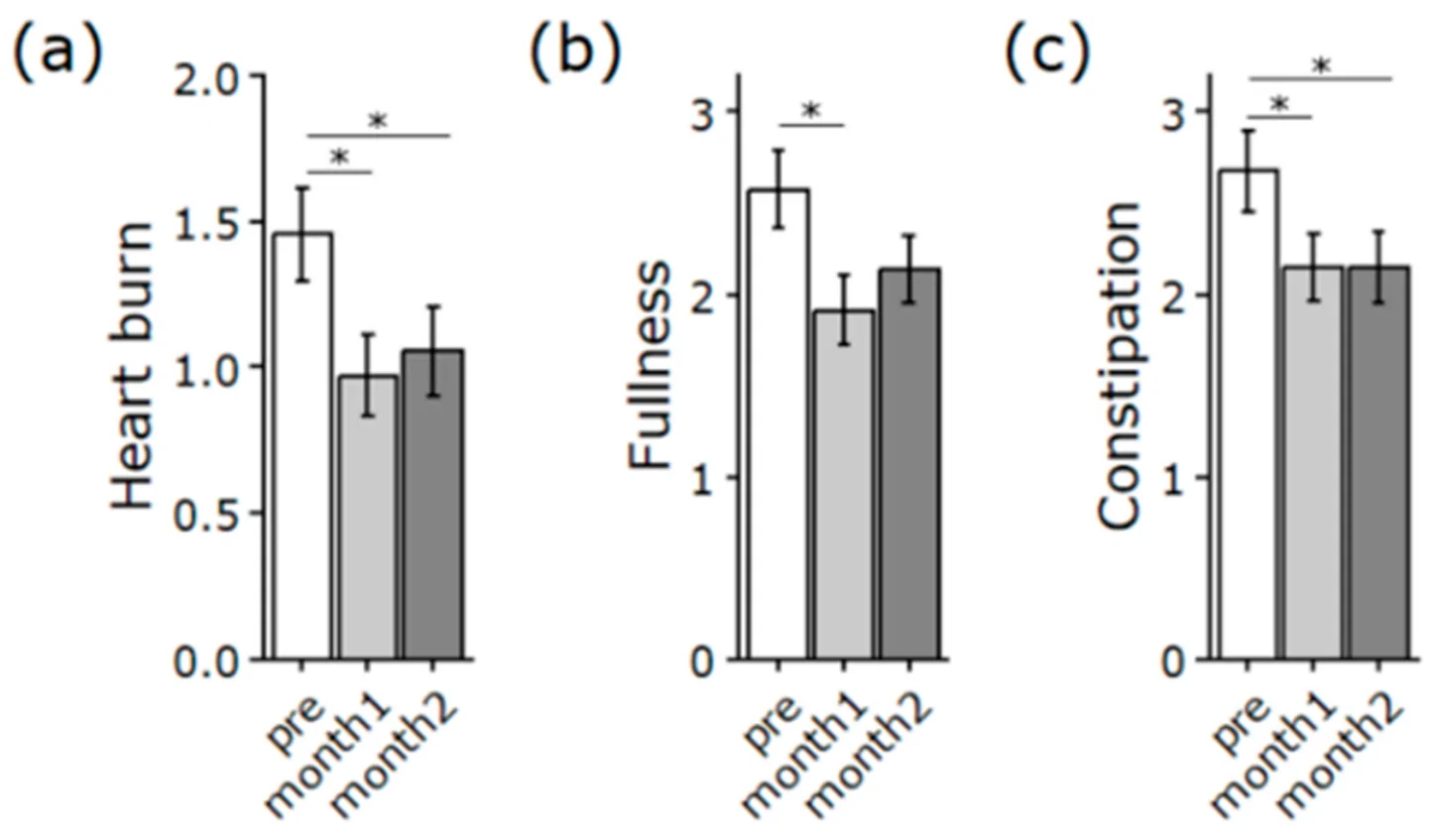
Scores of gastrointestinal symptoms based on the Izumo scale for (**a**) heart burn, (**b**) fullness, and (**c**) constipation. A higher score means a higher frequency of symptoms. * *p* < 0.05 by Tukey HSD after LMM.

Regarding gut microbiome diversity after two months of intervention, alpha-diversity analyses revealed that microbial richness (Chao1 index) remained unchanged (*p* > 0.05, Figure 2a), whereas evenness (Shannon index) was significantly decreased (*p* < 0.05, Figure 2b), concomitant with the expansion of specific genera. In terms of beta-diversity, principal coordinate analysis based on Bray–Curtis dissimilarity showed no statistically significant shift in overall microbial community structure across the total cohort (*p* > 0.05, Figure 2c). However, differential abundance analysis identified significant changes in 19 bacterial genera, characterized by increases in short-chain fatty acid (SCFA)-producing bacteria such as *Bifidobacterium* and *Faecalibacterium*, as well as *Prevotella*, which is involved in dietary fiber degradation (q < 0.10, Figure 2d). At baseline, the distribution of enterotypes was as follows: Type A (*n* = 11), Type B (*n* = 74), Type C (*n* = 13), and Type E (*n* = 23), with a predominance of Type B (Figure 2e). Following the intervention, 32% (39/121) of participants experienced a change in enterotype. Specifically, 45.8% (11/24) of participants with Type A or C shifted to Type B or E. Conversely, 12.4% (12/97) with Type B or E at baseline shifted to Type A, C, or D. Regarding digestive symptoms, subjective symptoms of heartburn and constipation significantly improved both after one and two months of intervention (*p* < 0.05, Figure 3a,b, Supplementary Table S2). Symptoms of indigestion significantly decreased at one month (*p* < 0.05), but showed no significant difference at two months despite remaining lower than baseline. (Figure 3c).

### 3.3. Labor Productivity and Sleep Quality

**Figure 4:**
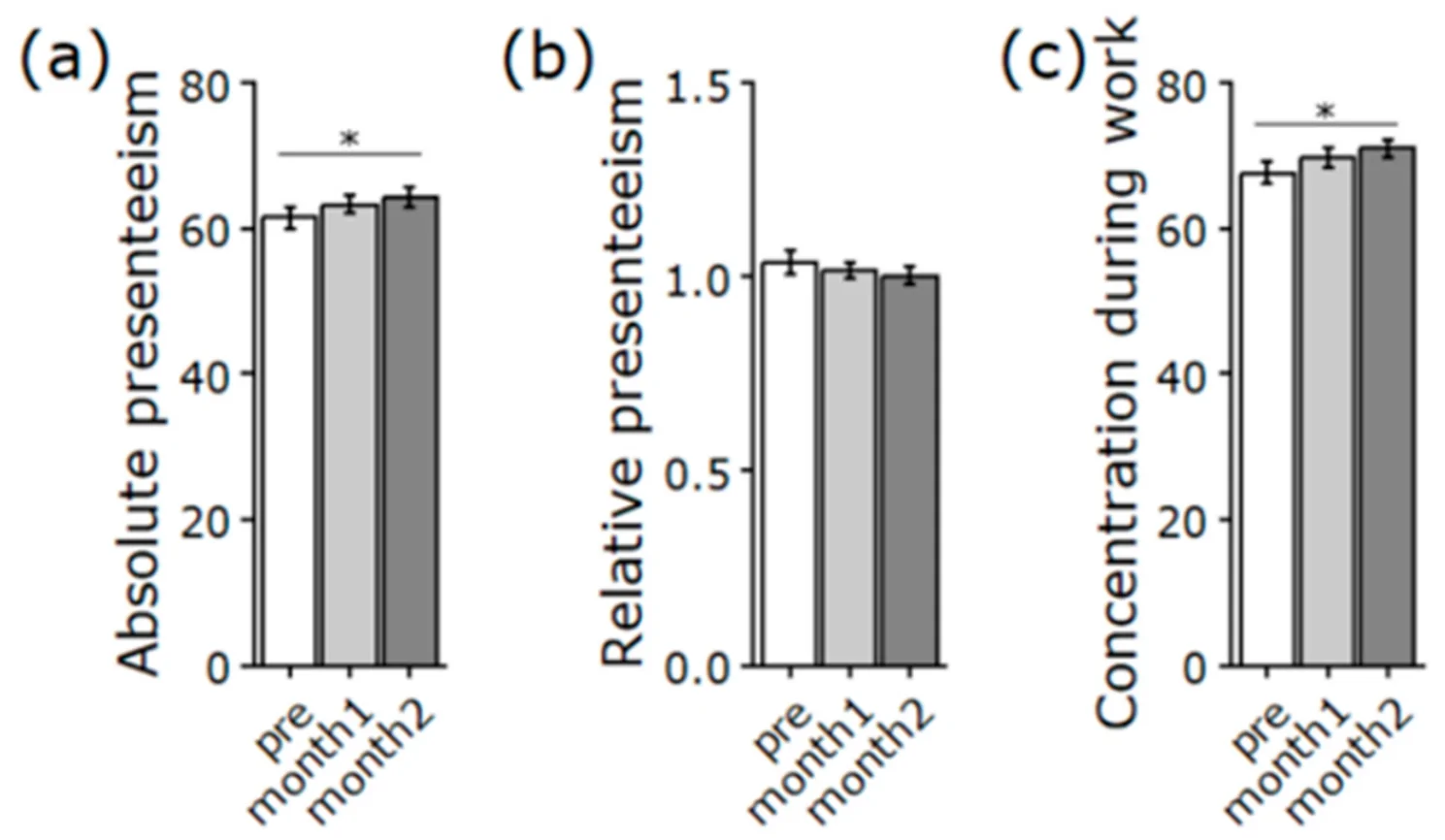
Indicators related to labor productivity. (**a**) Absolute presenteeism and (**b**) relative presenteeism from WHO-HPQ. (**c**) Subjective feeling about concentration during work. * *p* < 0.05 by Tukey HSD after LMM.

Presenteeism was evaluated based on the WHO-HPQ (Figure 4a,b). While no significant change was observed in relative presenteeism, absolute presenteeism significantly improved after two months of intervention (*p* < 0.05). Subjective evaluation of work-related items showed a significant improvement in concentration during work after two months (*p* < 0.05, Figure 4c, Supplementary Table S3). Regarding sleep quality assessed by the OSA-MA, “sleepiness on rising” significantly improved among the five factors (*p* < 0.05, Table 2). Other factors, except for “frequent dreaming,” showed numerical improvements, although these were not statistically significant (Table 2).

### 3.4. Correlation Analysis

**Figure 5:**
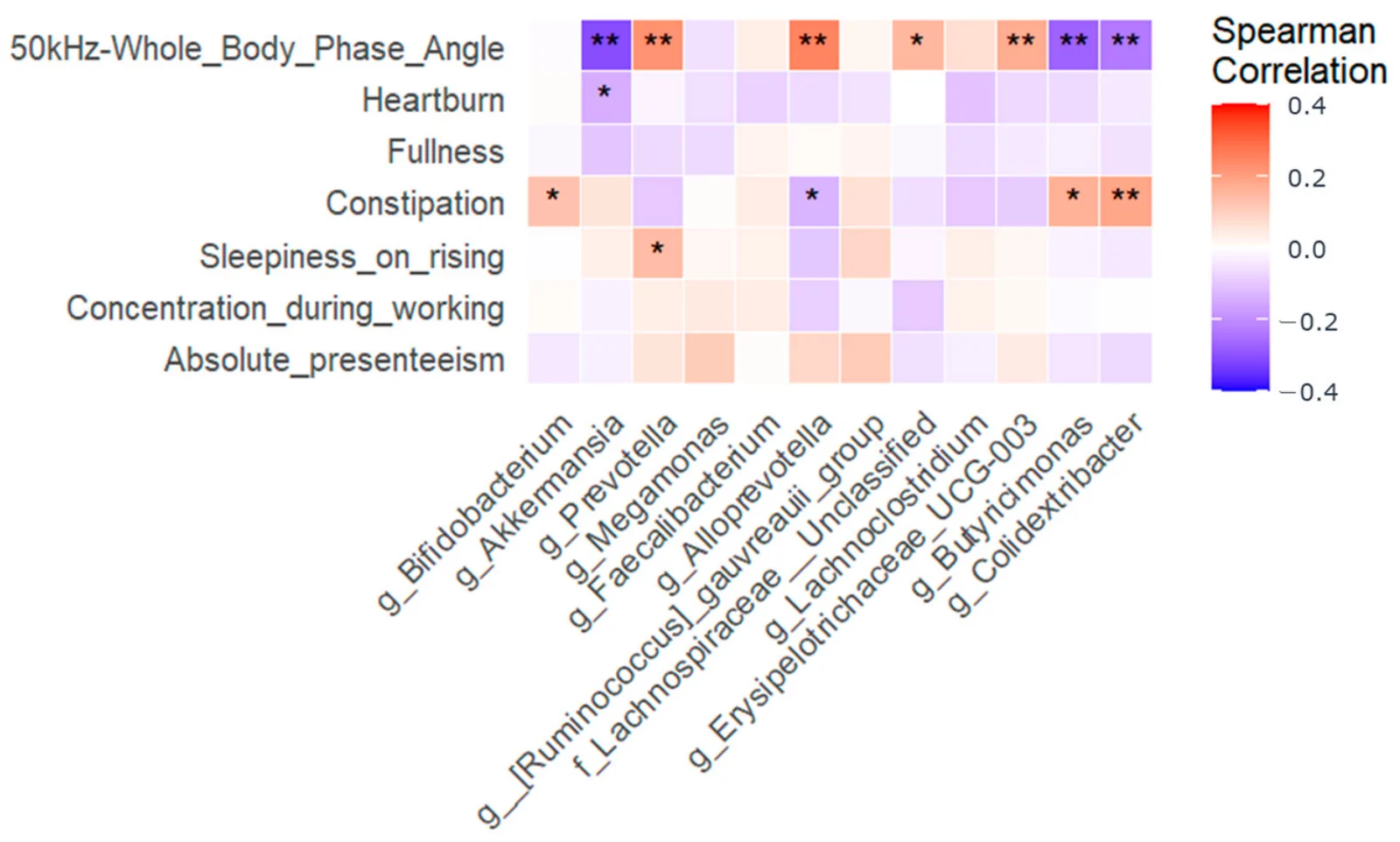
Spearman’s correlation between bacterial relative abundance and study outcomes. * *p* < 0.05 and ** *p* < 0.01.

Figure 5 presents the correlations between study outcomes and bacterial genera with a mean abundance > 0.1% that exhibited significant changes in relative abundance. Changes in PhA showed significant correlations with seven bacterial genera, suggesting a close link to gut microbiome dynamics. However, the bacteria enriched by the PHGG intervention, such as *Akkermansia*, did not necessarily exhibit positive correlations. Aside from fullness, concentration during working, and absolute presenteeism, which showed no significant associations, the other three outcomes also displayed significant correlations with specific bacterial genera. These correlation analyses were conducted strictly to explore potential associations and do not imply causal relationships.

### 3.5. Enterotype-Specific Responses to PHGG Intervention

While details of the baseline differences are provided in the Supplementary Materials, we confirmed distinct enterotype-specific characteristics concerning gut microbiome diversity, dietary habits, and some clinical parameters (Supplementary Figures S1 and S2). Given these baseline variations, we conducted exploratory subgroup analyses to observe longitudinal trends within each enterotype. It should be noted that the analysis for Types A, C, and E may lack sufficient statistical power due to the small sample sizes, as Type B accounted for the majority (61.2%) of the subjects. Regarding gut microbiota diversity, a significant decrease in the Shannon index was observed in Types A and B after intervention, and significant changes in beta-diversity were also confirmed in Type B (Supplementary Figure S3). No significant changes in these indices were observed in Types C and E. Regarding survey items, Type A showed a significant improvement in phase angle at month1 and values at month2 remained higher than baseline, though not significant (Table 3). Similarly, heartburn symptoms in Type A significantly improved at month1. Type B showed significant improvements in heartburn and constipation (Izumo scale), sleepiness on rising (OSA-MA), and absolute presenteeism. Type C showed significant improvement in indigestion, which was frequent at baseline. Type E, characterized by softer stools, showed a significant decrease in the Bristol Stool Scale score after two months, approaching the ideal score of 4.

## 4. Discussion

Novel insights from this exploratory study suggest that favorable shifts in the gut environment observed during continuous PHGG supplementation were accompanied by slight changes in bioimpedance parameters and positive changes in labor productivity parameters in Japanese office workers. Furthermore, our results suggest that the health-promoting effects may vary depending on the enterotype, and that continuous PHGG intake may induce enterotype transitions, although these subgroup findings remain purely exploratory.

Regarding the primary endpoint, the intervention induced specific genus-level alterations rather than a global shift in overall microbial community structure, as reflected by the absence of significant beta-diversity shifts (*p* > 0.05). In terms of alpha-diversity, while microbial richness remained stable, community evenness decreased, resulting in a lower Shannon index. Crucially, this reduction in the Shannon index should not automatically be interpreted as an “improvement” or an adverse dysbiosis. Rather, because species richness was preserved, this change mathematically reflects a temporary decrease in community evenness resulting from the rapid, selective proliferation of target prebiotic-utilizing genera—such as *Bifidobacterium* and *Faecalibacterium*—which dominated the community profile [24,25]. This aligns with previous findings that PHGG serves as a specific substrate for these taxa, leading to their dominance and a consequent reduction in diversity indices [26,27]. Furthermore, the analysis of enterotypes revealed a shift from Types A (*Coprococcus*, *Gemmiger*, *Roseburia* and *Ruminococcaceae* unclassified genera-dominant) and C (*Megamonas*, *Fusobacterium* and *Proteus*-dominant), which are often associated with dysbiosis or higher disease risks in the Japanese population, to Types B (*Bacteroides*, *Blautia* and *Faecalibacterium*-dominant) and E (*Prevotella*-dominant) [13]. According to large-scale cohort studies by Takagi et al., Type A and Type C in the Japanese population are more highly associated with specific diseases such as functional gastrointestinal disorders [13]. Nevertheless, a shift away from disease-associated enterotypes toward Type B or Type E should be interpreted with caution. Enterotypes represent complex, highly dynamic ecological classifications, and their relationships with health outcomes remain strictly context-dependent. Furthermore, epidemiological associations with specific enterotypes do not directly establish that changing an individual’s enterotype leads to clinical health improvements. Specifically, Type E, characterized by *Prevotella* abundance, is typically associated with habitual high dietary fiber intake, suggesting that PHGG supplementation may, at least partly, mimic a high-fiber dietary pattern [28]. While enterotypes are generally considered resilient to short-term dietary modifications [29,30,31], certain prebiotic interventions have been reported to influence microbial community structures [21]. The enterotype transitions observed in this study provide preliminary, exploratory evidence of microbiome responsiveness to continuous PHGG intake, although further rigorous, well-powered studies are required for confirmation.

A notable physiological observation was the significant, albeit small, increase in PhA without concomitant changes in body weight or skeletal muscle mass. PhA is an indirect parameter derived from bioelectrical impedance analysis that broadly reflects cell membrane capacitance, tissue hydration, and cellular integrity [32]. Although lower PhA values have been associated with cellular damage, inflammation, or poor clinical prognosis in diseased populations [32,33,34], PhA is an indirect measurement that is susceptible to various physiological dynamics, including acute fluctuations in hydration status and fluid distribution. Therefore, the observed increase in PhA in this healthy office worker cohort should be interpreted with caution and cannot be presented as direct biological evidence of improved “cellular health” or “cellular integrity” caused by PHGG supplementation. Rather, PhA in this study represents a non-invasive surrogate metric whose change may mirror broader, aggregate physiological shifts during the intervention period. Although the literature exploring the relationship between the gut microbiome and bioimpedance parameters remains limited, the significant correlations observed between PhA changes and specific bacterial genera warrant further mechanistic investigation [35,36]. It is also interesting to note that PhA levels are low in enterotypes Type A and C, which are associated with higher disease prevalence.

Regarding labor productivity, favorable changes were observed in absolute presenteeism during the supplementation period, accompanied by concurrent improvements in sleep quality and various digestive symptoms, including constipation and upper gastrointestinal symptoms such as heartburn and indigestion. PHGG supplementation has previously been associated with improvements in constipation [14], sleep quality [15,26], and motivation [26], which were concurrently observed alongside enhanced labor productivity parameters in the present study. However, there have been no previous reports suggesting the efficacy of PHGG on upper gastrointestinal function. Therefore, our findings regarding the alleviation of these upper gastrointestinal symptoms represent a novel potential health benefit of PHGG, although further verification is required. PHGG has also been reported to improve not only constipation but also loose stools [16], and its efficacy in improving loose stool tendencies in Type E suggests broad applicability of PHGG across the population. Comparisons based on enterotypes revealed differences in baseline gastrointestinal symptoms and post-intervention subjective experiences across types. This suggests that interventions targeting diverse populations with mixed health statuses like a company, require either individualized approaches or interventions with broad efficacy. In this regard, despite substantial statistical power limitations in smaller enterotype subgroups, these stratified exploratory observations provide preliminary, hypothesis-generating insights regarding baseline gut microbiota variability in corporate populations, which require confirmation in larger, balanced cohorts. In correlation analyses, changes in specific bacterial abundance did not necessarily correspond to all observed health benefits. For instance, while absolute presenteeism showed an overall significant improvement after the intervention, it displayed no significant correlations with any single bacterial genus. This suggests that certain complex health benefits, such as labor productivity, may not be fully explained by single-taxa fluctuations alone. Instead, they might be mediated by collective shifts in the overall microbial community structure or the aggregate production of their metabolites, such as short-chain fatty acids. Additionally, the high diversity of subject backgrounds, with enterotypes being a key contributing factor, may have further complicated the detection of these taxon-level correlations. Furthermore, we emphasize that these observed associations are strictly correlational and do not establish direct causality between specific bacterial shifts and clinical or productivity improvements.

The potential biological pathways underlying the observed changes in subjective health and labor productivity during PHGG supplementation remain speculative. Although prebiotics such as PHGG are well-documented to enhance short-chain fatty acid (SCFA) production, suppress systemic inflammation and influence the central nervous system via the gut–brain axis [15,37,38,39,40], we did not measure fecal or circulating SCFA concentrations, inflammatory cytokines, or metabolic biomarkers in the present trial. Consequently, any involvement of SCFA-mediated gut–brain axis pathways in our findings remains entirely hypothetical. These proposed biological mechanisms should be interpreted strictly as speculative hypotheses to guide future mechanistic studies rather than verified pathways in our trial cohort.

## 5. Limitation

Several limitations of this study warrant mention.

First, the open-label, single-arm design is a major limitation that prevents establishing direct causality. Without a randomized placebo-control group, the observed improvements in subjective metrics like presenteeism cannot be definitively attributed to PHGG supplementation. These changes may have been influenced by non-specific factors, including placebo responses, regression to the mean, the Hawthorne effect, or voluntary behavioral shifts in personal health habits. Second, the pre-specified exclusion of participants with severe obesity (BMI ≧ 35) to avoid broad physiological and analytical confounding introduces a minor selection bias. Consequently, our findings should be primarily generalized to healthy, non-severely obese office workers. Third, this study was conducted during the winter months (January to March), coinciding with a period when workloads typically increase toward the end of the fiscal year. This seasonal factor may have introduced bias regarding physical condition. Fourth, dietary intake was monitored only at baseline. We cannot rule out the possibility that participants voluntarily altered their background diet during the intervention period. Fifth, due to the uneven distribution of subjects across enterotypes and small sample sizes in Types A, C and E, these subgroup analyses suffered from limited statistical power and an inherent multiple-testing burden. Consequently, all enterotype-specific responses must be viewed as exploratory, hypothesis-generating observations that require confirmation in larger, well-powered studies. In summary, because this preliminary single-arm trial cannot rule out placebo effects, natural symptom fluctuations, or background dietary shifts, these findings should be interpreted strictly as hypothesis-generating. Future rigorous randomized, placebo-controlled trials incorporating longer follow-up periods, continuous dietary monitoring, and objective biological markers (such as fecal short-chain fatty acids and systemic inflammatory cytokines) are essential to validate these preliminary observations.

## 6. Conclusions

In conclusion, this preliminary study demonstrates that daily supplementation with PHGG for two months was accompanied by positive shifts in the gut microbiome, gastrointestinal symptoms, sleep quality, and labor productivity parameters in office workers. The observed shifts in enterotypes and the slight increase in whole-body phase angle suggest overall positive trends in physiological and subjective parameters during the study period. While a single-arm design cannot rule out placebo or non-specific effects, these findings provide hypothesis-generating evidence that continuous PHGG supplementation may serve as a practical dietary strategy in workplace settings, warranting confirmation in future randomized placebo-controlled trials.

## Figures and Tables

**Table 1:** Subject characteristics and change in body composition.

Characteristics	Pre (*n* = 136)				Month1 (*n* = 110)				Month2 (*n* = 125)			
age	48.37	±	0.76			-				-		
Height (cm)	167.05	±	0.73			-				-		
Body weight (kg)	72.84	±	1.24		72.58	±	1.35		72.40	±	1.27	
Body Mass Index	25.93	±	0.32		25.94	±	0.36		25.85	±	0.33	
Body fat mass (kg)	22.78	±	0.58		22.85	±	0.67		22.61	±	0.61	
Body fat ratio (%)	31.14	±	0.56		31.21	±	0.63		31.08	±	0.60	
Muscle mass (kg)	47.17	±	0.85		46.86	±	0.88		46.92	±	0.87	
Skeletal Muscle Mass (kg)	27.64	±	0.54		27.51	±	0.56		27.42	±	0.55	
Bone Mineral Content (kg)	2.89	±	0.05		2.87	±	0.05		2.87	±	0.05	
Total Body Water (L)	36.74	±	0.66		36.51	±	0.68		36.46	±	0.68	
Protein (kg)	9.83	±	0.18		9.78	±	0.18		9.75	±	0.18	
Minerals (kg)	3.49	±	0.06		3.48	±	0.06		3.46	±	0.06	
50 kHz-Whole Body Phase Angle (degree)	5.58	±	0.06		5.67	±	0.06	*	5.61	±	0.06	*

Data are expressed as mean ± SE; * *p* < 0.05 from baseline (pre).

**Table 2:** OSA-MA scores.

Factor	Pre (*n* = 125)				Month1 (*n* = 132)				Month2 (*n* = 130)			
Sleepiness on rising	16.25	±	0.53		17.71	±	0.53	*	17.90	±	0.52	*
Initiation and maintenance of sleep	18.77	±	0.53		19.26	±	0.55		19.04	±	0.52	
Frequent dreaming	23.82	±	0.66		23.49	±	0.65		22.55	±	0.69	
Refreshing on rising	15.74	±	0.56		16.24	±	0.54		16.58	±	0.57	
Sleep length	15.89	±	0.60		17.02	±	0.59		16.57	±	0.61	

Data are expressed as mean ± SE; * *p* < 0.05 from baseline (pre).

**Table 3:** Survey items that significantly changed within the study period in each gut microbiome enterotype.

Items	Pre				Month1				Month2			
TypeA												
50 kHz-Whole Body Phase Angle (degree)	4.99	±	0.18		5.14	±	0.14	*	5.11	±	0.18	
Heart burn	0.64	±	0.28		0.09	±	0.09	*	0.18	±	0.18	
TypeB												
Heart burn	1.58	±	0.24		1.00	±	0.19	*	1.19	±	0.22	
Constipation	2.70	±	0.31		1.96	±	0.20	*	1.97	±	0.26	*
Absolute presenteeism	58.90	±	2.19		63.78	±	1.69	*	64.59	±	1.73	*
Sleepiness on rising	15.97	±	0.71		17.80	±	0.70	*	18.53	±	0.62	*
TypeC												
Indigestion	4.33	±	1.05		1.85	±	0.71	*	1.85	±	0.53	*
TypeE												
Bristol scale	4.78	±	0.27		4.39	±	0.28		4.09	±	0.31	*
50 kHz-Whole Body Phase Angle (degree)	5.79	±	0.10		6.03	±	0.10		5.92	±	0.11	*

Data are expressed as mean ± SE; * *p* < 0.05 from baseline (pre).

## Data Availability

The 16S rRNA gene sequencing data generated and analyzed during this study have been deposited in the NCBI Sequence Read Archive repository under BioProject accession number PRJNA1521603 (BioSample accession numbers SAMN62805569 to SAMN62805810).

## Supplementary Materials

The following supporting information can be downloaded at: https://www.mdpi.com/article/10.3390/microorganisms14102215/s1, Figure S1: Chao1 and Shannon indices of each enterotype gut microbiome at baseline (pre); Figure S2: Baseline characteristics for each enterotype; Figure S3: Change in gut microbial alpha (Chao1 and Shannon) and beta diversity in each enterotype; Table S1: Comparison of baseline characteristics between participants who completed and missed the Month1 assessment; Table S2: Scores of gastrointestinal symptoms (Based on Izumo-scale); Table S3: Subjective feelings related to labor productivity.
