# Supplementary material for: Preliminary Evaluation of Dietary Partially Hydrolyzed Guar Gum on Work Motivation and Quality of Life of Office Workers: An Open-Label, Single-Arm Study
Source: Microorganisms. 2026 Oct 2;14(10):2215. doi: 10.3390/microorganisms14102215 (PMC13707965; doi:10.3390/microorganisms14102215)
Supplement: Supplementary file 1 [file microorganisms-14-02215-s001.zip › microorganisms-4506364-supplementary.pdf]

Supplementary Tables

Supplementaly table S1. Comparison of baseline characteristics between participants who completed and missed the Month1 assessment.

	Characteristics	Month1 Completers (n = 110)	Month1 Non-completers (n = 26)	p-value
Body composition	age	48.55 ± 0.87	47.62 ± 1.52	0.63
	Height (cm)	166.78 ± 0.76	168.17 ± 2.12	0.46
	Body weight (kg)	72.58 ± 1.35	73.91 ± 3.10	0.68
	Body Mass Index	25.94 ± 0.36	25.87 ± 0.65	0.93
	Body fat mass (kg)	22.79 ± 0.67	22.75 ± 1.20	0.98
	Body fat ratio (%)	31.14 ± 0.62	31.13 ± 1.32	0.99
	Muscle mass (kg)	46.92 ± 0.89	48.18 ± 2.43	0.56
	Skeletal Muscle Mass (kg)	27.50 ± 0.57	28.25 ± 1.54	0.59
	Bone Mineral Content (kg)	2.87 ± 0.05	2.98 ± 0.14	0.40
	Total Body Water (L)	36.55 ± 0.69	37.55 ± 1.89	0.56
	Protein (kg)	9.78 ± 0.19	10.03 ± 0.51	0.59
	Minerals (kg)	3.47 ± 0.06	3.60 ± 0.18	0.42
	50kHz-Whole Body Phase Angle (degree)	5.61 ± 0.07	5.45 ± 0.12	0.29
Digestive symptoms	Heartburn	1.49 ± 0.18	1.29 ± 0.32	0.63
	Fullness	2.46 ± 0.23	3.08 ± 0.53	0.27
	Constipation	2.75 ± 0.25	2.36 ± 0.44	0.50
Labor productivity	Absolute presenteeism	61.85 ± 1.50	59.57 ± 4.52	0.56
	Relative presenteeism	1.01 ± 0.02	1.16 ± 0.12	0.06
	Concentration during work over the past four weeks	52.08 ± 2.10	41.70 ± 5.55	0.43
	Sleepiness during work over the past four weeks	67.05 ± 1.51	70.13 ± 4.42	0.38
	Motivation for work over the past four weeks	69.21 ± 1.57	73.70 ± 3.79	0.25
	Fatigue over the past four weeks	58.75 ± 2.83	52.61 ± 6.77	0.05
Sleep quality	Sleepiness on rising	16.16 ± 0.58	16.66 ± 1.08	0.72
	Initiation and maintenance of sleep	18.62 ± 0.56	19.45 ± 1.23	0.55
	Frequent dreaming	23.81 ± 0.70	23.86 ± 1.50	0.97
	Refreshing on rising	15.82 ± 0.58	15.41 ± 1.34	0.78
	Sleep length	15.52 ± 0.62	17.59 ± 1.45	0.19

Data are expressed as mean ± SE; No statistically significant differences were observed (p ≥ 0.05).

Supplementary table S2. Scores of gastrointestinal symptoms (Based on Izumo-scale).

Category	Factor	pre (n =131)	month1 (n =133)	month2 (n =130)
Heartburn	Are you bothered by acid reflux?	0.56 ± 0.08	0.33 ± 0.06 *	0.31 ± 0.06 *
	Are you bothered by heartburn centered in the anterior chest?	0.25 ± 0.05	0.16 ± 0.04	0.27 ± 0.06
	Are you bothered by throat discomfort?	0.65 ± 0.09	0.48 ± 0.08	0.47 ± 0.08
	SUM	1.45 ± 0.16	0.97 ± 0.14 *	1.05 ± 0.15 *
Gastralgia	Are you bothered by epigastric pain?	0.55 ± 0.08	0.33 ± 0.06 *	0.44 ± 0.08
	Are you bothered by an epigastric burning sensation?	0.27 ± 0.06	0.11 ± 0.03 *	0.19 ± 0.05
Fullness	Are you bothered by early satiation?	0.76 ± 0.08	0.61 ± 0.07	0.72 ± 0.08
	Are you bothered by post-prandial long-lasting epigastric fullness or nausea?	0.79 ± 0.09	0.52 ± 0.07 *	0.56 ± 0.07 *
	Are you bothered by epigastric bloating?	1.05 ± 0.10	0.78 ± 0.09 *	0.85 ± 0.09
	SUM	2.58 ± 0.22	1.91 ± 0.19 *	2.14 ± 0.18
Constipation	Are you bothered by a feeling of incomplete defecation?	1.44 ± 0.10	1.22 ± 0.09	1.18 ± 0.09 *
	Are you bothered by constipation or hard stools?	0.78 ± 0.10	0.59 ± 0.08 *	0.62 ± 0.08
	Are you bothered by stress-related cinstipation?	0.50 ± 0.08	0.34 ± 0.06	0.34 ± 0.06
	SUM	2.68 ± 0.22	2.15 ± 0.19 *	2.15 ± 0.20 *
Diarrhea	Are you bothered by fecal urgency?	1.00 ± 0.10	0.89 ± 0.10	0.85 ± 0.09
	Are you bothered by stress-related diarrhea?	0.48 ± 0.07	0.42 ± 0.06	0.34 ± 0.06

Data are expressed as mean ± SE; *p <0.05 from baseline (pre).

Supplementaly table S3. Subjective feelings related to labor productivity

question	pre (n =131)	month1 (n =133)	month2 (n =131)
Concentration during work over the past four weeks	67.59 ± 1.49	69.68 ± 1.31	70.92 ± 1.24 *
Sleepiness during work over the past four weeks	57.67 ± 2.67	56.63 ± 2.66	60.31 ± 2.55
Motivation for work over the past four weeks	70.00 ± 1.48	68.36 ± 1.51	71.25 ± 1.44
Fatigue over the past four weeks	50.26 ± 2.05	50.37 ± 1.95	51.63 ± 2.12

Values are subjectively evaluated by subjects from 0 (min) to 100 (max). Data are expressed as mean ± SE; *p <0.05 from baseline (pre).

Supplementary Materials

Enterotype-specific characteristics in the present study

Regarding the gut environment, a recent report by Takagi *et al.* indicates that the gut microbiome of the Japanese population can be broadly classified into five enterotypes.(13) While associations between these types and dominant bacterial genera or diseases have been documented, the distribution of these enterotypes among healthy office workers, their relationship with general health status, and the differential effects of interventions on specific gut microbiota profiles remain largely unexplored.

We examined differences in baseline characteristics among enterotypes. Alpha-diversity indices (Chao1 and Shannon indices) were significantly higher in Type A compared to other enterotypes (Supplementary Figure S1). PCoA based on Bray-Curtis dissimilarity showed that each enterotype formed significantly distinct unique clusters ($p < 0.05$, Supplementary Figure S2a). Dietary intake analysis, a major factor forming enterotypes, suggested that Type A had significantly higher calcium and cholesterol intake compared to other enterotypes, possibly due to habitual consumption of eggs and milk (Supplementary Figure S2b). Comparison of baseline survey items among enterotypes revealed that Type E had significantly higher Bristol Stool Scale scores compared to Types A and C, indicating a tendency toward loose stools (Supplementary Figure S2c). Indigestion symptoms on the Izumo scale were most frequent in Type C ($p < 0.05$, Supplementary Figure S2c). The phase angle was significantly lower in Type A compared to Types B and E, suggesting potential issues with cellular health ($p < 0.05$). Regarding labor productivity evaluated by VAS, Type B reported significantly worse fatigue over the past four weeks compared to Type C ($p < 0.05$).

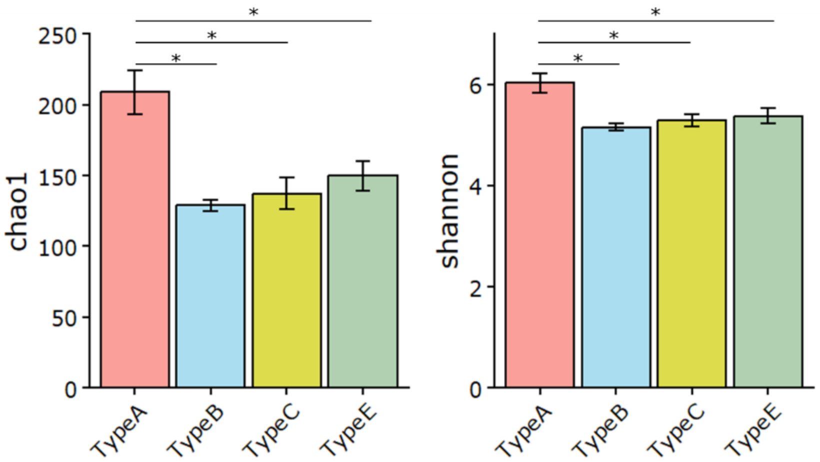

Supplementary Figure S1. Chao1 and Shannon indices of each enterotype gut microbiome at baseline (pre). *$p < 0.05$ by Tukey HSD after LM.

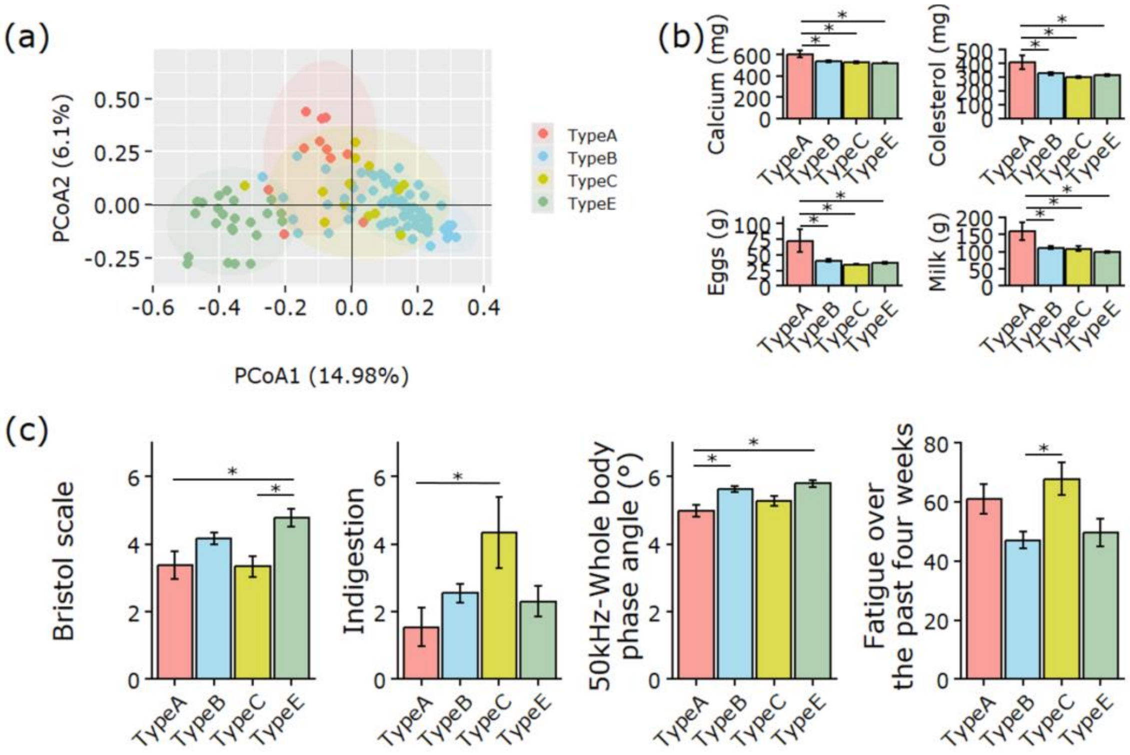

Supplementary Figure S2. Baseline characteristics for each enterotype. (a) Principal coordination analysis based on the Bray–Curtis dissimilarity index. The ellipse enclosing each cluster indicates a 95% confidence interval. $p < 0.05$ between every enterotype by pairwise permutational ANOVA test. Bonferroni-corrected p-values were calculated by the R package pairwiseAdonis. Nutritional status (b) and study outcomes (c) were significantly different between enterotypes. *$p < 0.05$ by Tukey HSD after LM.

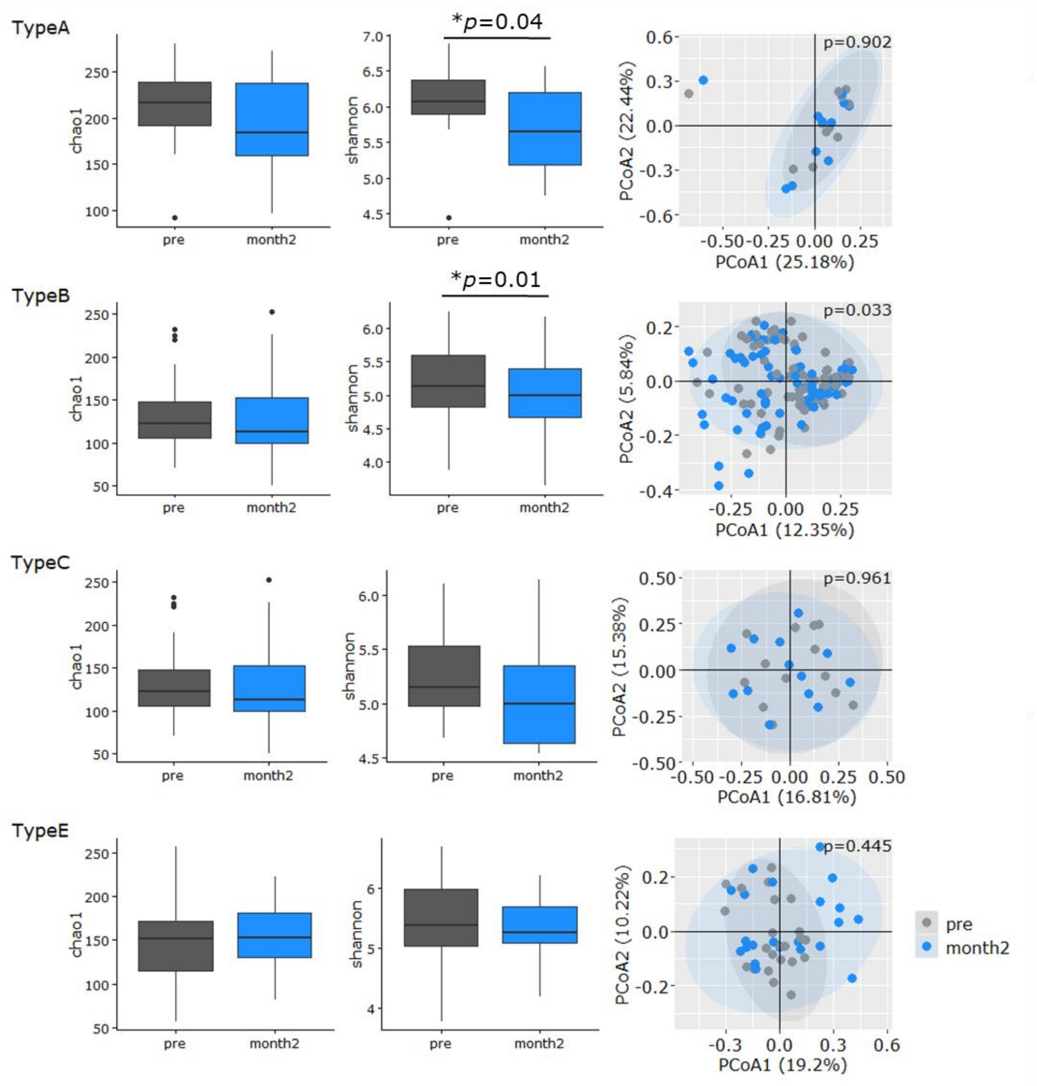

Supplementary Figure S3. Change in gut microbial alpha (Chao1 and Shannon) and beta diversity in each enterotype. Statistical significance was evaluated using LMM for alpha diversity and by PERMANOVA for beta diversity.
